# Sacro-anterior haemangiopericytoma: a case report

**DOI:** 10.7497/j.issn.2095-3941.2014.02.010

**Published:** 2014-06

**Authors:** Xiu-Hong Ge, Shuai-Shuai Liu, Hu-Sheng Shan, Zhi-Min Wang, Qian-Wen Li

**Affiliations:** ^1^Department of Graduate, Bengbu Medical College, Bengbu 233000, China; ^2^Cancer Center, No. 82 Hospital of People’s Liberation Army Subsidiary, Huai’an 223001, China

**Keywords:** Sacro-anterior, haemangiopericytoma (HPC), soft tissue tumor

## Abstract

Haemangiopericytoma (HPC) is a rare vascular tumor with borderline malignancy, considerable histological variability, and unpredictable clinical and biological behavior. HPC can present a diagnostic challenge because of its indeterminate clinical, radiological, and pathological features. HPC generally presents in adulthood and is equally frequent in both sexes. HPC can arise in any site in the body as a slowly growing and painless mass. The precise cell type origin of HPC is uncertain. One third of HPCs occur in the head and neck areas. Exceptional cases of hemangioblastoma arising outside the head and neck areas have been reported, but little is known about their clinicopathologic and immunohistochemical features. This study reports on a case of a large sacro-anterior HPC in a 65-year-old male.

## Introduction

Haemangiopericytoma (HPC) is an extraordinarily rare soft tissue tumor. HPC originates from the pericytes of blood vessels, which is a specific cell type that surrounds the capillary vessels. Therefore, HPC can occur in areas where capillaries are found. HPC was first named and described in 1942 by Stout and Murray^1^^,^^2^. The most frequent locations of HPC are the extremities, pelvis, retroperitoneum, head, neck, and meninges. HPCs are usually deeply located within the muscle tissue, but dermal and subcutaneous masses have also been described^3^. According to the literature, approximately one-third of all HPCs occur in the head and neck^4^. No HPC cases were found in the sacro-anterior. The pathogenesis of HPC is unclear and its clinical features are non-specific and vary according to the location of the tumor^5^. The incidence of HPC is highest among individuals aged 50 to 60 years old. The diagnosis of HPC depends on histopathologic and immunohistochemistry (IHC) findings. HPC can be classified as benign, borderline, and malignant depending on its histopathologic and clinical features^6^^,^^7^. Not all HPCs are typical; some cases of HPC present overlapping features with other vascular and mesenchymal tumors. IHC helps in excluding other differential diagnoses, but some cases with unusual findings can pose a challenge to general pathologists^3^.

## Case report

**Figure 1:**
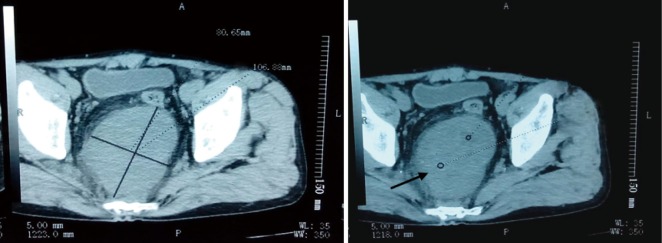
Abdominal CT: two pre-operation images of the abdomen. From the images, we can observe a large quasi-circular tumor, which is about 10.7 cm × 8.1 cm in the sacro-anterior. The tumor is intensified asymmetrically and its boundary is clear. The surrounding fat gap of the tumor is clear and no lymph node was found. CT, computerized tomography.

**Figure 2:**
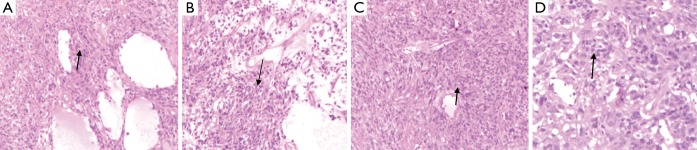
These four images illustrate the pathology of the sacro-anterior tumor. (A-C) Cancer cells involves spindle cell or oval cell and there are pericyte in perivascular. Cancer cells growth-arranged by radialted, concentric or irregular (H&E staining ×10); (D) there are deckzelles which are rounded, ovate or fusiform in perivascular, there are mitotic index in some nuclear (H&E staining ×40).

A 65-year-old male underwent a computerized tomography (CT) scan because of abdominal pain. The non-contrast-enhanced CT scan revealed a space-occupying lesion approximately 10.7 cm × 8.1 cm in the sacro-anterior. The tumor was asymmetrical and its boundary was clear. The surrounding fat gap of the tumor was clear and no enlarged lymph gland was found (**Figure 1**). However, the malignancy of the tumor remained undetermined. One week later, a radical surgical excision was attempted in a different hospital. The post-operative pathology revealed that the tumor was a sacro-anterior hemanyiopericytoma. Immunohistochemical staining of the tumor revealed CD34 (+++), S-100 (–), SMA (–), EMA (–), CD117 (–), Bcl-2 (+-++), CD99 (++), NF-(L+H) (–), MyoD1 (++), and Ki-67 (40%+) (**Figure 2**).

**Figure 3:**
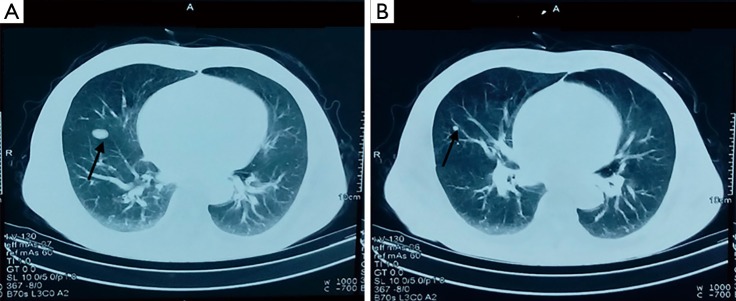
Two chest CT images show HPC metastasis in the right lung. The larger metastatic tumor is located in the middle and lower lobe of the anterior basal segment. The tumor is approximately 1.8 cm × 1.1 cm with smooth morphology (A). The smaller tumor is about 0.5 cm × 0.6 cm (B). CT, computerized tomography; HPC, haemangiopericytoma.

**Figure 4:**
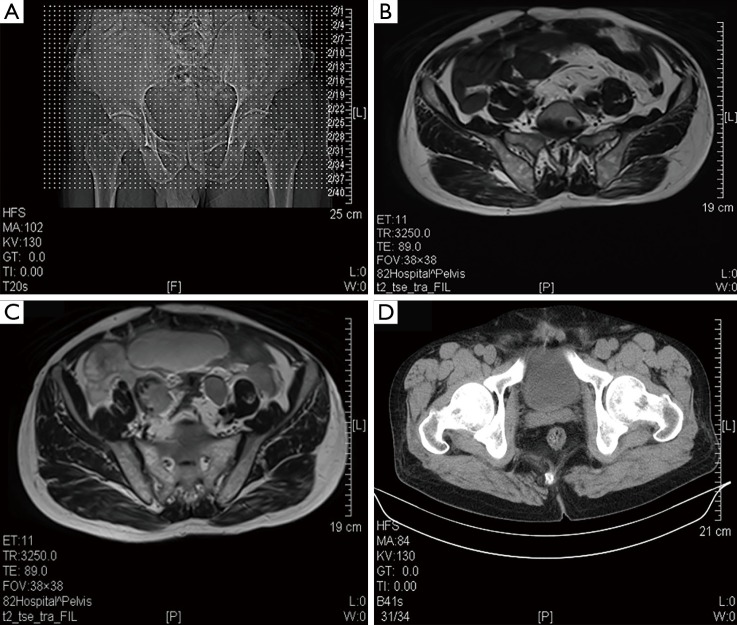
Three abdominal CT images taken 6 months after the last cycle of EP chemotherapy showed no signs of HPC recurrence. CT, computerized tomography; HPC, haemangiopericytoma.

After the tumor was excised, the patient reported no pain or discomfort. Hence, he refused chemoradiotherapy. Ten months later, the patient felt abdominal pain. The patient then went to the hospital and underwent color Doppler ultrasound of the pelvic cavity. The ultrasound results revealed that the HPC recurred, resulting in the patient receiving radical surgical excision again. The pathology and IHC certified that the excised tumor was a recurred HPC. However, the patient still refused chemoradiotherapy. One month later, the patient suffered from frequent coughing and then underwent a chest CT scan. The chest CT scan revealed that the tumor had metastasized into the right lung. The larger metastatic tumor is located in the middle and lower lobe of the anterior basal segment. The tumor was approximately 1.8 cm × 1.1 cm and had a smooth morphology. The smaller tumor was approximately 0.5 cm × 0.6 cm (**Figure 3**). The patient accepted five cycles of EP (VP-16 + DDP) chemotherapy and metastases radiotherapy. After chemoradiotherapy, the patient’s condition improved. Six months later, the patient had no complaints and the CT scan showed no evidence of recurrence (**Figure 4**). The study was approved by the institutional ethics committee.

## Discussion

While the classification scheme of the World Health Organization (WHO) for CNS neoplasms considers SFT and HPC as distinct entities with different prognostic implications^8^, SFT and HPC are considered variants of a single entity, with the term “HPC” considered as obsolete^8^. Recent detection of NAB2-STAT6 fusions in both SFT and HPC has provided further support to the notion of a single pathologic entity^9^^,^^10^.

HPC is a rare vascular tumor that originates from the pericytes of blood vessels that are also called Zimmurman cells^11^. The Zimmurman cell is a discontinuous spindle cell, which adheres to the reticular fiber membrane. The Zimmurman cell is a juvenile mesenchymal cell that facilitates contraction and potential secretion and can transfer to HPC under pathologic conditions^12^. The peak prevalence age of HPC is 50 to 60 years old, primarily in adult life and at the median age of 55 years regardless of gender^13^^-^^15^. HPC has a propensity for recurrence and metastasis and is frequently aggressive. HPC can occur anywhere in the body. About one-third of all HPCs occur in the head and neck, while others in extremities, pelvis, retroperitoneum, and meninges. Primary sacro-anterior HPCs are very rare tumors and only a few cases have been reported. A painless mass is the most common presentation of head and neck HPCs^7^. The clinical behavior of HPC varies depending on the tumor grade. A definitive diagnosis of HPC is provided by accurate histopathologic assessment and cannot be made on the basis of clinical and imaging findings [e.g., CT and magnetic resonance imaging (MRI)]^16^. Imaging examinations can only judge the size and boundary of the tumor. Previous studies reported that HPCs observed by CT generally appear as well-circumscribed masses, which are dramatically enhanced after injection of contrast material; meanwhile in MRI images, HPCs are highly intense in T1 and slightly intense in T2^3^. The general morphology of the tumor is round to oval-shaped, and the size of the tumor ranges from 5 to 15 cm^17^, with or without diolame^18^.

HPC has no pathognomonic clinical or radiological signs. Therefore, diagnosis can only be made after careful histopathological examination. The diagnosis of HPC is often made after excluding other tumors that demonstrate HPC-like features. The histologic characteristics of HPC are the tumors that have rich capillary networks and closely arranged tumor cells in the peripheral blood vessels. Tumor cells are intensive skin cells that may be round, oval, or fusiform with unclear boundaries^5^^,^^6^. The relative characteristics of HPC immunohistochemically are CD34, Vimentin, CD99, and BCL-2 positive and negative Actim and Desmin^19^. The prediction of the clinical behavior of HPC does not always correlate with the histolopathologic features of the tumor. The strict universal histopathologic criteria for malignancy have not been made. The existing malignancy criteria for HPC vary between different studies. The most common metastases sites of HPC are the lungs, mediastinum, liver, and bone. Unfortunately, the aggressive behavior of HPC is unpredictable because benign tumors can become malignant and metastasize^20^. Generally, the large size (>5 cm), increased mitotic rate (>4 mitosis/10 H PF), presence of atypical mitosis, high cellularity, pleomorphic tumor cells, and foci of hemorrhage and necrosis predict a highly malignant course^7^^,^^13^^-^^15^. A tumor that has a size larger than 2.0 cm and located in a deep site (in the absence of nuclear atypia) demonstrates uncertain malignant potential^20^.

The histopathology of HPCs has been well-documented, but the fine-needle aspiration biopsy (FNAB) results are rarely described^21^^-^^24^. FNAB is difficult to use in the diagnosis of primary HPC. However, cytologic analysis may diagnose the recurrence or metastasis of HPC^23^. Incisional biopsy for tumors is not advised because the tumor is considered to be vascular, and the risk of bleeding and consequent fibrosis is present. Furthermore, FNAB is not used in HPC because the primary treatment is always surgery regardless of the pre-operative cytologic findings.

Given the unexpected and potentially aggressive behavior of HPCs, complete excision is the recommended treatment. The common problem with HPC is local recurrence after incomplete excision, with rates varying from 13% to 40%. In cases of local recurrence, adjuvant radiotherapy and chemotherapy can be offered.

Although the results of adjuvant radiotherapy and chemotherapy vary, adjuvant radiotherapy and chemotherapy are still used as supplementary treatment for HPCs. HPCs are not sensitive to radiotherapy. Radiotherapy is reserved only as adjuvant therapy for cases of incompletely excised tumors, recurrent tumors, and tumors with high-grade histopathologic features. Chemotherapy may have a role in the treatment of distant metastatic disease, but its role in the primary treatment of HPC remains unclear. Vincristine, Adriamycin, and Cyclophosphamide have been used in cases of aggressive HPCs with variable success^25^. Some reports showed that the use of interferon may benefit patients with pulmonary HPC metastases^26^. Some studies reported local recurrence rates of as high as 40%, with metastatic disease in 15% of the cases reported with a latency period ranging from 63 to 107 months^27^. In our case, after operation, radiotherapy, and chemotherapy, the patient’s clinical symptoms nearly completely disappeared, and the CT scan (**Figure 4**) revealed that the tumor did not recur.

## Conclusion

HPC is a rare soft tissue tumor with high histological variability and unpredictable clinical and biological behavior. One third of HPCs occur in the head and neck and only a few cases have been reported regarding tumor localization in the sacro-anterior. Pre-operative evaluation must include a thorough imaging evaluation (CT and MRI scan), including the results that may not be specific for HPCs. The treatment of choice is radical excision. HPCs are potentially malignant, regardless of the benign pathology. Given the possibility of recurrence and metastasis in HPC cases, surgical excision may not be sufficient^28^. After tumor excision, adjuvant chemotherapy is necessary. During follow-up visits, imaging examination is used to determine recurrence and metastasis.

## References

[1] Stout AP Murray MR Hemangiopericytoma: a vascular tumor featuring Zimmermann’s pericytes. Ann Surg 1942;116:26-33 17858068 10.1097/00000658-194207000-00004 PMC1543753

[2] Carvalho JR Haddad L Leonhardt FD Marques Filho MF Santos Rde O Cervantes O Head and neck hemangiopericytoma in a child: case report. Sao Paulo Med J 2004;122:223-226 15558145 10.1590/S1516-31802004000500010 PMC11160341

[3] Pacheco LF Fernandes BF Miyamoto C Maloney SC Arthurs B Burnier MN Jr Rapid growth of an orbital hemangiopericytoma with atypical histopathological findings. Clin Ophthalmol 2014;8:31-33 24353402 10.2147/OPTH.S47901 PMC3862697

[4] Russell WO Cohen J Enzinger F Hajdu SI Heise H Martin RG A clinical and pathological staging system for soft tissue sarcomas. Cancer 1977;40:1562-1570 907970 10.1002/1097-0142(197710)40:4<1562::aid-cncr2820400428>3.0.co;2-6

[5] Sun Y Tian JH Li M Shi CJ Two cases of hemangiopericytoma. Journal of Chinese Clinical Oncology 2010,15:284-285

[6] McMaster MJ Soule EH Ivins JC Hemangiopericytoma. A clinicopathologic study and long-term followup of 60 patients. Cancer 1975;36:2232-2244 1203874 10.1002/cncr.2820360942

[7] Enzinger FM Smith BH Hemangiopericytoma. An analysis of 106 cases. Hum Pathol 1976;7:61-82 1244311 10.1016/s0046-8177(76)80006-8

[8] Zambo I, Veselý K. WHO classification of tumours of soft tissue and bone 2013: the main changes compared to the 3rd edition. Cesk Patol 2014;50:64-70. 24758500

[9] Chmielecki J Crago AM Rosenberg M O’Connor R Walker SR Ambrogio L Whole-exome sequencing identifies a recurrent NAB2-STAT6 fusion in solitary fibrous tumors. Nat Genet 2013;45:131-132 23313954 10.1038/ng.2522 PMC3984043

[10] Schweizer L Koelsche C Sahm F Piro RM Capper D Reuss DE Meningeal hemangiopericytoma and solitary fibrous tumors carry the NAB2-STAT6 fusion and can be diagnosed by nuclear expression of STAT6 protein. Acta Neuropathol 2013;125:651-658 23575898 10.1007/s00401-013-1117-6

[11] Zimmermann KW Der feinere Bau der Blutcapillaren. Zeitschrift fur Anatomie und Entwicklungsgeschichte 1923;68:29-109

[12] Ma XX Chen BH Intraspinal haemangiopericytoma: two cases report and literature review. Chinese Bone and Joint Surgery 2009;2:87-91

[13] Billings KR Fu YS Calcaterra TC Sercarz JA Hemangiopericytoma of the head and neck. Am J Otolaryngol 2000;21:238-243 10937909 10.1053/ajot.2000.8378

[14] Ghaffar H Parwani A Rosenthal DL Fine needle aspiration cytology of hepatic metastasis from a meningeal hemangiopericytoma. A case report. Acta Cytol 2003;47:281-286 12685202 10.1159/000326517

[15] Chhieng D Cohen JM Waisman J Fernandez G Cangiarella J Fine-needle aspiration cytology of hemangiopericytoma: A report of five cases. Cancer 1999;87:190-195 10455206 10.1002/(sici)1097-0142(19990825)87:4<190::aid-cncr5>3.0.co;2-y

[16] Middleton LP Duray PH Merino MJ The histological spectrum of hemangiopericytoma: application of immunohistochemical analysis including proliferative markers to facilitate diagnosis and predict prognosis. Hum Pathol 1998;29:636-640 9635686 10.1016/s0046-8177(98)80015-4

[17] Lan RQ Wang ZC Hemangiopericytoma and pathological diagnosis of relative tumor. Journal of Clinical and Experimental Pathology 2012;28:595-599

[18] Feng Y Cheng GX Liu T Zhang G Imaging diagnosis of hemangiopericytoma. J South Med Univ 2009;29:1046-1068

[19] Zhang X Xu WJ Gao CP One case of pterygopalatine fossa hemangiopericytoma. Acta Academiae Medicinae Qingdao Universitatis 2010,46;182

[20] Jo VY Fletcher CD WHO classification of soft tissue tumours: an update based on the 2013 (4th) edition. Pathology 2014;46:95-104 24378391 10.1097/PAT.0000000000000050

[21] Katsantonis GP Friedman WH Rosenblum BN The surgical management of advanced malignancies of the parotid gland. Otolaryngol Head Neck Surg 1989;101:633-640 2512551 10.1177/019459988910100604

[22] Sawh RN Lele SM Borkowski J Ventura KC Zaharopoulos P Logroño R Fine-needle aspiration cytology of hemangiopericytoma: report of two cases. Diagn Cytopathol 2000;23:187-191 10945907 10.1002/1097-0339(200009)23:3<187::aid-dc9>3.0.co;2-y

[23] Geisinger KR Silverman JF Cappellari JO Dabbs DJ Fine-needle aspiration cytology of malignant hemangiopericytomas with ultrastructural and flow cytometric analyses. Arch Pathol Lab Med 1990;114:705-710 2363628

[24] Shimizu K Ogura S Kobayashi TK Kushima R Toyokuni S Iwasa Y Fine-needle aspiration cytology of malignant hemangiopericytoma of the salivary gland: A case report. Diagn Cytopathol 1999;21:398-401 10572271 10.1002/(sici)1097-0339(199912)21:6<398::aid-dc6>3.0.co;2-v

[25] Lackner H Urban C Dornbusch HJ Schwinger W Kerbl R Sovinz P Interferon alfa-2a in recurrent metastatic hemangiopericytoma. Med Pediatr Oncol 2003;40:192-194 12518351 10.1002/mpo.10122

[26] Carew JF Singh B Kraus DH Hemangiopericytoma of the head and neck. Laryngoscope 1999;109:1409-1411 10499045 10.1097/00005537-199909000-00009

[27] Guthrie BL Ebersold MJ Scheithauer BW Shaw EG Meningeal hemangiopericytoma: histopathological features, treatment, and long-term follow-up of 44 cases. Neurosurgery 1989;25:514-522 2797389

[28] Palacios E Restrepo S Mastrogiovanni L Lorusso GD Rojas R Sinonasal hemangiopericytomas: clinicopathologic and imaging findings. Ear Nose Throat J 2005;84:99-102 15794546

